# Volatilomic Differentiation of Protected‐Origin Italian Balsamic Vinegars by HS‐SPME‐GC×GC‐TOFMS

**DOI:** 10.1002/jssc.70442

**Published:** 2026-05-11

**Authors:** Sofia Malcangi, Allan Polidoro, Monica Romagnoli, Alberto Cavazzini, Flavio A. Franchina

**Affiliations:** ^1^ Department of Chemical, Pharmaceutical, and Agricultural Sciences University of Ferrara Ferrara Italy; ^2^ Department of Environmental and Prevention Sciences University of Ferrara Ferrara Italy

**Keywords:** Balsamic vinegar, comprehensive two‐dimensional gas chromatography, food authenticity, GC×GC‐TOFMS, HS‐SPME, volatilomics

## Abstract

Balsamic vinegars produced under Protected Designation of Origin (PDO) and Protected Geographical Indication (PGI) schemes differ substantially in raw materials, processing intensity, and aging regimes. Although these differences are expected to shape their volatile composition, a comprehensive chemical characterization capable of reliably discriminating the two denominations remains analytically challenging. In this study, headspace solid‐phase microextraction combined with comprehensive two‐dimensional gas chromatography‐time‐of‐flight mass spectrometry (HS‐SPME‐GC×GC‐TOFMS) was used to characterize PDO and PGI balsamic vinegars and to evaluate the feasibility of volatilomic differentiation between the investigated products. Extraction conditions were comparatively assessed using a pooled quality‐control sample by evaluating two SPME fiber coatings (PDMS/DVB and DVB/CAR/PDMS) and a high ionic‐strength condition (40% *w/v* NaCl); the DVB/CAR/PDMS fiber combined with salting‐out provided the highest analytical response and was selected for subsequent analyses. After peak detection and alignment, 1397 features were initially detected and reduced to 227 after blank and artifact filtering. Mann‐Whitney U testing with false discovery rate correction identified 100 statistically significant features, of which 67 were putatively identified. Multivariate analysis revealed a clear separation between PDO and PGI samples: analyzed PDO vinegars were enriched in furanoids, lactones, esters, aldehydes, acids, phenols, ketones, and alcohols, whereas PGI samples showed comparatively higher levels of terpenoids. These chemical trends align with the technological differences between the two production systems. Mapping volatile features to literature‐reported odor descriptors suggested that the higher abundance of furanoids, lactones, and related compounds in the PDO samples may contribute to sweeter, caramel‐like, creamy, balsamic, woody, and floral nuances. Overall, the integration of HS‐SPME‐GC×GC‐TOFMS with statistical filtering and multivariate analysis demonstrates the feasibility of a volatilomic analytical workflow capable of differentiating selected PDO and PGI products, and establishes a methodological framework for future large‐scale studies on authenticity and quality control.

## 1 Introduction

Balsamic vinegars (BVs) from the Emilia‐Romagna region of northern Italy are internationally recognized for their complex aroma, rich flavor, and deep cultural roots. Their production involves thermal concentration of the must, alcoholic and acetic fermentations, and maturation in wooden barrels, which together determine the product's chemical and sensory characteristics [1, 2, 3, 4, 5, 6].

Owing to their historical relevance, artisanal production practices, and economic value, regulatory frameworks were developed to define production standards and protect region‐specific manufacturing methods formally. Within this context, two types of balsamic vinegar are recognized under European quality schemes designed to safeguard foods with specific geographical and technological characteristics. These frameworks, Protected Designation of Origin (PDO) and Protected Geographical Indication (PGI), aim to maintain the authenticity of traditional products, regulate production standards, and protect them from imitation or misuse of name. Accordingly, two types of BV are recognized within these frameworks: *Aceto Balsamico Tradizionale di Modena* (ABTM), which holds the PDO label, and *Aceto Balsamico di Modena* (ABM), which has the PGI label. Although both are produced within the same geographical area, their specifications differ substantially in terms of raw materials, fermentation dynamics, and aging procedures [5, 6, 7, 8, 9].

The PDO product, ABTM, is obtained exclusively from cooked grape must sourced within the provinces of Modena and Reggio Emilia. The must undergoes natural alcoholic and acetic fermentations and is subsequently matured for a minimum of twelve years (and at least 25 years for the “*extravecchio*” category) in a series of wooden barrels of decreasing volume made from different types of wood. The aging process involves annual refilling (*rincalzo*) and proceeds under seasonal temperature fluctuations, promoting progressive concentration, oxidation, and esterification, which give the traditional product its characteristic dark color, syrupy consistency, and complex aroma. The addition of wine vinegar, caramel, or any other ingredient is not permitted under the PDO specification [5, 6, 8].

In contrast, ABM, protected by a PGI, is produced under a regulated but more flexible and industrial process. It is obtained by blending partially fermented, cooked, or concentrated grape must with wine vinegar. The mixture must contain at least 20% grape must and 10% wine vinegar, and may include up to 2% caramel for color adjustment. It is aged for a comparatively short time in wooden vessels within the provinces of Modena or Reggio Emilia for at least 60 days, or for at least 3 years to qualify for the “*invecchiato*” designation. The PGI specification allows greater flexibility in raw‐material proportions and maturation duration, resulting in products with greater variability in viscosity, color, and aromatic complexity than traditional PDO vinegars [5, 6, 7].

Because of these contrasts and the high economic relevance of both denominations, verifying product authenticity represents an analytical challenge. Conventional physicochemical and sensory assessments retain their value for quality control but provide limited chemical information. Given that PDO and PGI vinegars share similar raw materials, fermentation pathways, and volatile matrices, such approaches cannot capture the subtle compositional variations arising from differences in must processing and aging dynamics. Consequently, more comprehensive analytical strategies are required to characterize their chemical profiles and support reliable authentication [3, 5, 10, 11].

Headspace solid‐phase microextraction (HS‐SPME) has become a widely adopted sample‐preparation technique for the study of (semi‐)volatile organic compounds in complex matrices. Its solvent‐free nature, ease of automation, and ability to preconcentrate analytes directly from the HS make it particularly suited for food aroma analysis. In BVs, HS‐SPME enables simultaneous extraction of compounds spanning a broad polarity and volatility range, while minimizing matrix interferences and thermal degradation [3, 5, 12, 13, 14].

Comprehensive two‐dimensional gas chromatography coupled to time‐of‐flight mass spectrometry (GC×GC‐TOFMS) provides the separation capacity required to resolve the highly complex volatile fraction of BVs. The combination of two columns with different stationary phases enhances peak capacity and minimizes coelution, enabling detailed profiling of BV constituents [15, 16]. When combined with HS‐SPME, GC×GC‐TOFMS offers a powerful analytical strategy for the comprehensive characterization of volatile compounds from BVs.

In this context, the present study employs HS‐SPME coupled with GC×GC‐TOFMS to investigate the volatile composition of selected PDO and PGI balsamic vinegars. Through univariate and multivariate analyses, the study aims to identify volatile features statistically associated with differences between the analyzed PDO and PGI samples, relate these compounds to literature‐reported odor descriptors, and examine how differences in production and aging may be reflected in characteristic compositional patterns within the volatilome.

## 2 Experimental Section

### 2.1 Vinegar Samples

A total of five commercial balsamic vinegar samples were analyzed, two of which comprised products labeled as “*Aceto Balsamico Tradizionale di Modena*” (PDO) and three “*Aceto Balsamico di Modena*” (PGI). All samples were obtained from certified local producers and commercial suppliers. The PDO and PGI designations were verified according to the official product labeling and European Union certification marks. Upon arrival at the laboratory, the samples were stored in the dark at room temperature (∼23°C). Before extraction, each sample was homogenized and aliquoted (250 µL) into 20 mL glass vials.

A pooled quality control (QC) sample was prepared by mixing equal volumes of all individual samples to represent the average volatilomic profile of the dataset. The QC sample was used to evaluate the extraction parameters and chromatographic separation and to assess instrumental repeatability during the sample analysis batch.

### 2.2 HS‐SPME Procedure

Volatile compounds were extracted from BV samples by HS‐SPME using a PAL autosampler (CTC Analytics AG, Zwingen, Switzerland). All experiments were carried out using screw‐cap 20 mL glass vials fitted with PTFE/silicone septa. For each assay, 250 µL of the sample was aliquoted into the vials. The vials were equilibrated at 40°C for 5 min under agitation (1100 rpm), after which the SPME fiber was exposed to the headspace for 40 min at the same temperature. At the end of the extraction step, injections were performed using an Optic‐4 multimode injector (GL Sciences B.V., Eindhoven, Netherlands) equipped with an inlet Peltier cooler. The SPME fiber was introduced into the GC×GC inlet, initially at 45°C, then ramped to 270°C at 10°C s^−1^, where it was maintained for 2 min for thermal desorption in split mode (1:60).

In order to evaluate the influence of fiber coating on the extraction performance, Polydimethylsiloxane/Divinylbenzene (PDMS/DVB, 65 µm) and Divinylbenzene/Carboxen/Polydimethylsiloxane (DVB/CAR/PDMS, 50/30 µm) fibers (Supelco, Bellefonte, USA) were compared under identical conditions. The fibers were conditioned prior to use according to the manufacturer's recommendations. Each fiber was tested in triplicate (*n* = 3) using a QC sample and the aforementioned extraction protocol. Extraction performance was comparatively evaluated based on the analytical response (TIC) obtained from GC×GC‐TOFMS analysis. Differences between fibers were assessed using Student's t‐test at a 95% significance level.

The same experimental framework was subsequently used to assess the effect of matrix ionic strength on analytical response. Extractions of QC samples were performed in the absence and presence of analytical‐grade sodium chloride (NaCl, 40 % *w/v*), added directly to the vial and dissolved before equilibration. Both conditions (with and without NaCl) were analyzed in triplicate, and the chromatographic area obtained for each condition was assessed using Student's *t*‐test at a 95% significance level.

Based on these comparative evaluations, final HS‐SPME extractions for all subsequent analyses were performed using the DVB/CAR/PDMS fiber and 40% (*w/v*) NaCl.

To monitor analytical consistency, analytical internal standards, 2‐nitrotoluene and nitrobenzene (Restek, Bellefonte, USA), were spiked into each sample (10 µL of 1 µg mL^−1^ solution). Their response was evaluated against statistical thresholds: values below 2 standard deviations indicated acceptable variability, values between 2 and 3 standard deviations were flagged as a caution zone requiring further attention, and values above 3 standard deviations indicated a significant deviation that would compromise the analysis's reliability, requiring corrective actions. The corresponding control charts are provided in Figure S1.

### 2.3 Chromatographic Conditions

The GC×GC‐TOFMS analyses were performed on an Agilent 8890 GC, equipped with a PAL System device (CTC Analytics AG), coupled to a Pegasus BT 4D time‐of‐flight mass spectrometer (LECO Corporation, Mönchengladbach, Germany). Chromatographic separation was achieved using an Rxi‐5 ms column (30 m × 0.25 mm × 0.25 µm d*_f_*) and an Rxi‐17SilMS column (2 m × 0.25 mm × 0.25 µm d*_f_*) as primary (^1^D) and secondary (^2^D) dimensions, respectively (both from Restek Corporation, Bellefonte, PA, USA). Ultra‐high‐purity helium (99.999 %) was used as the carrier gas, at a flow rate of 1.5 mL min^−1^.

The GC oven temperature program began at 45°C (held for 2 min), followed by a ramp of 7°C min^−1^ to 240°C, and then a rapid ramp of 30°C min^−1^ to 320°C (held for 2 min). Temperature offsets for the secondary oven and modulator were set at +10°C and +15°C, respectively. A 3‐s modulation period was employed (hot jet 0.5 s; cold jet 1.0 s). The transfer line and electron ionization (EI) ion source temperatures were set at 280°C, with an ionization energy of 70 eV. A mass range of 40–500 *m/z* was collected, with an acquisition delay of 180 s and an acquisition frequency of 150 Hz. The data were collected using ChromaTOF software version 5.56 (LECO Corporation).

### 2.4 Data Processing and Statistical Analysis

Raw GC×GC‐TOFMS data were processed using ChromaTOF Sync v. 2.0.15.0 (LECO, St. Joseph, USA). Peak detection and spectral deconvolution were performed automatically by the software prior to alignment. The expected minimum ^2^D full width at half‐height (FWHH) threshold was set to 50 ms, and the S/N threshold to 50. Alignment was performed using retention‐time and spectral‐similarity mapping, with retention‐shift tolerances of 6.0 s in the first dimension and 0.1 s in the second dimension. The resulting aligned feature table was exported as a .csv file for subsequent statistical processing.

A univariate comparison between the PDO and PGI groups was performed using the Mann‐Whitney U test on the aligned features’ responses. Resulting p‐values were adjusted for multiple comparisons using the Benjamini‐Hochberg false discovery rate (FDR) procedure. Features with FDR‐adjusted p < 0.05 were considered statistically significant and retained for subsequent annotation and multivariate analyses.

Tentative compound annotation was performed by comparing spectral similarity and retention index (RI) values with the NIST23 library. Two confidence levels were established for compound identification: *Putative ID^i^* and *Putative ID^ii^*. For *Putative ID^i^*, annotations were accepted when both the mass spectral similarity score was ≥850, and the experimental RI matched the reference within ±20 units. For *Putative ID^ii^*, a lower threshold was applied, requiring spectral similarity ≥750 with the same RI tolerance (±20 units). The ^1^D retention indices were calculated using a C_7_‐C_30_ alkane series (Supelco, Bellefonte, USA), analyzed under the same conditions as the samples.

Principal component analysis (PCA) and hierarchical cluster analysis (HCA) were performed using Orange Data Mining software (version 3.39) [17]. Before multivariate analysis, peak areas of the significant features were autoscaled (mean‐centered and divided by the standard deviation) to minimize the influence of intensity range differences and to enhance comparability across variables. PCA was applied to obtain an unsupervised overview of variance structure and sample clustering. HCA was conducted using Euclidean distance and Ward's linkage to explore similarity patterns among samples and among volatile features. Heatmaps were generated to visualize abundance gradients across chemical classes.

## 3 Results and Discussion

Prior to sample analysis, HS‐SPME conditions were evaluated using a pooled QC sample to ensure adequate chemical coverage and analytical repeatability. The comparison of fiber coatings (PDMS/DVB vs. DVB/CAR/PDMS) and the assessment of salt addition (40% *w/v* NaCl) are detailed in the Supplementary Material (Section S1). Briefly, DVB/CAR/PDMS, combined with salting‐out, yielded a higher overall response and improved repeatability and was therefore selected for all subsequent analyses.

### 3.1 Feature Selection

**FIGURE 1:**
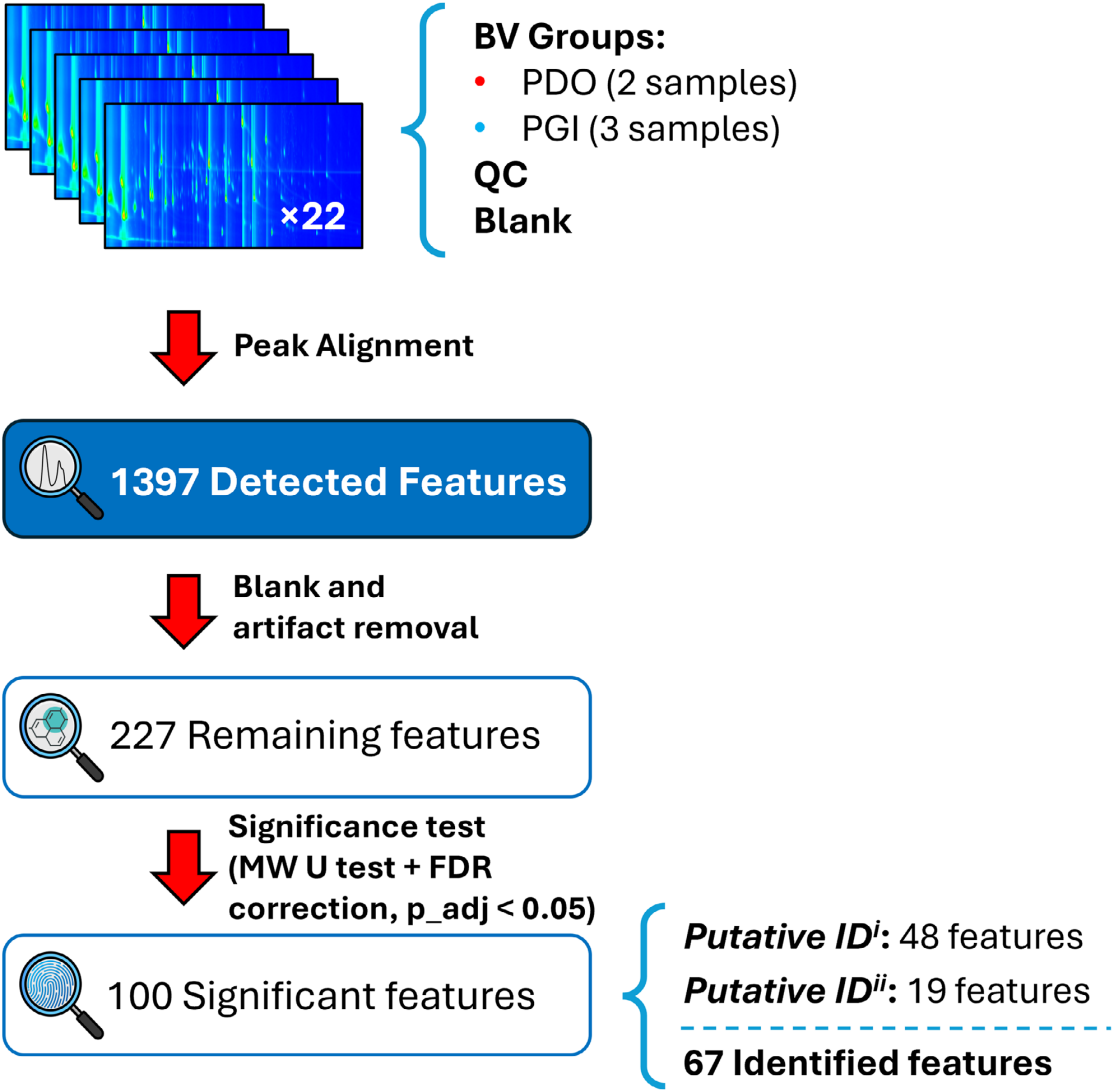
Workflow for feature detection, alignment, filtering, and identification of Balsamic vinegar (BV) volatiles.

After establishing the HS‐SPME conditions and acquiring the complete GC×GC‐TOFMS dataset for the BV, the next step was to select features suitable for comparative analysis between the PDO and PGI sample groups. Given the complexity of the BV volatilome and the large number of peaks detected by GC×GC‐TOFMS, a structured filtering workflow was applied to retain only chemically meaningful and statistically reliable signals. This process included automated peak detection, removal of artifacts and blank‐related features, and statistical screening based on group comparisons. The overall feature‐selection pipeline is summarized in Figure 1, which illustrates the progressive reduction from the initial aligned feature set to the final list of significant variables, which are then subjected to identification and chemometric interpretation.

The complete data matrix consisted of 22 chromatograms: PDO samples (2 products × 3 replicates), PGI samples (3 products × 3 replicates), a pooled QC sample analyzed in six replicates, and a blank injection. After chromatographic alignment across the runs, the software detected 1397 features, reflecting the inherent chemical complexity of BV and the enhanced resolution power and high peak capacity of the GC×GC‐TOFMS technique.

Since this initial list includes noise‐related peaks, column bleed, and other non‐sample artifacts, a blank subtraction and artifact‐filtering step was applied. This procedure reduced the dataset to 227 remaining features, representing signals detected in real samples while absent or negligible in blanks, ensuring that subsequent statistical evaluation was restricted to chemically reliable features.

To determine which volatiles were statistically associated with differences between the analyzed PDO and PGI sample groups, a Mann‐Whitney U test was performed on the aligned feature table. The resulting *p*‐values were adjusted for multiple comparisons using the Benjamini‐Hochberg FDR procedure. This screening identified 100 significant features (FDR‐adjusted *p* < 0.05), forming a subset of statistically significant features within the present dataset. Of these, 67 were putatively identified based on mass spectral similarity and RI agreement (Table S1). Among them, 48 were identified under the *Putative ID^i^* criteria (match factor > 850; ΔRI < 20), while 19 compounds were identified under the *Putative ID^ii^* criteria (match factor > 750; ΔRI < 20).

**FIGURE 2:**
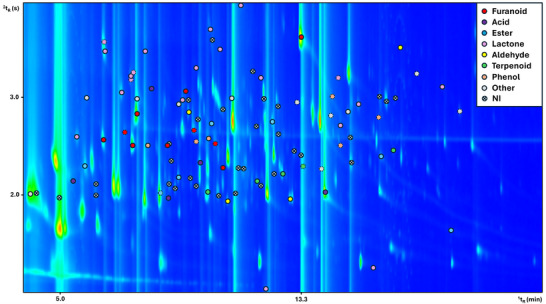
Comprehensive two‐dimensional gas chromatography coupled to time‐of‐flight mass spectrometry (GC×GC‐TOFMS) chromatogram of a quality control (QC) sample showing the features retained after false discovery rate (FDR)‐controlled Mann‐Whitney U testing (Benjamini‐Hochberg correction; FDR‐adjusted *p* < 0.05) that differentiate between the analyzed Protected Designation of Origin (PDO) and Protected Geographical Indication (PGI) samples. Colored circles indicate compounds according to the chemical class listed in the top right corner. Full‐lined circles correspond to analytes identified under the *Putative ID^i^* criteria, while dashed‐lined circles represent analytes identified under the *Putative ID^ii^* criteria. NI features (marked with crossed circles) represent statistically significant peaks that did not meet the identification thresholds.

The GC×GC‐TOFMS chromatogram of a QC sample shown in Figure 2 provides an overview of the volatilomic complexity of BV, illustrating the chromatographic distribution of the discriminant features retained following univariate screening. The second‐order separation offered by the GC×GC technique distributes compounds reflecting their volatility (^1^D) and polarity (^2^D), generating characteristic patterns that facilitate the visualization of structurally related chemical classes. Against this structured separation, the overlaid colored circles represent the subset of volatiles that are statistically associated with the observed differences between the PDO and PGI samples analyzed.

Each marker on the chromatogram corresponds to a statistically significant feature. Their dispersion across the chromatographic plane illustrates the wide variety of physicochemical properties represented among the discriminant compounds, revealing the analytical advantage of comprehensive two‐dimensional separation: compounds that would likely coelute in one‐dimensional GC appear as fully resolved peaks, enabling confident detection and identification. This visualization also underscores the heterogeneity of the BV matrix, in which discriminant compounds span from multiple chemical domains, from highly volatile species detected early in the ^1^D to less volatile, polar analytes appearing later and at higher ^2^D retention times.

### 3.2 Multivariate Analysis

In order to explore the structure of the volatilomic dataset, unsupervised multivariate analyses were applied to features that significantly differentiated PDO and PGI samples, enabling simultaneous evaluation of covariation patterns and revealing sample‐level relationships and compound groupings that cannot be captured by univariate tests alone.

PCA was first employed to provide an unsupervised overview of sample clustering and major sources of variance. HCA and heatmap visualization were subsequently used to examine feature‐level abundance patterns and to assess the consistency of the separation observed in PCA.

**FIGURE 3:**
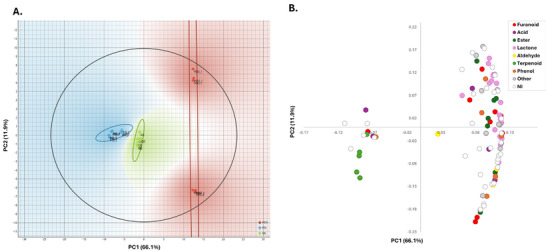
Principal component analysis (PCA) of balsamic vinegar samples based on volatile compound profiles. (A) Score plot showing Protected Designation of Origin (PDO), Protected Geographical Indication (PGI), and quality control (QC) samples with Hotelling's T^2^ confidence ellipses (95%). (B) Loading plot indicating the contribution of each significant feature to the first two principal components, colored according to chemical class.

Principal component analysis (Figure 3) provided a global overview of the data structure, with the first two components accounting for 78% of the total variance. In the score plot (Figure 3A), PDO, PGI, and QC samples formed distinct and compact clusters, with tight grouping of replicates and non‐overlapping Hotelling's T^2^ confidence ellipses (95%), demonstrating satisfactory within‐group reproducibility and clear discrimination among sample types. The main separation occurred along PC1, with PGI samples clustering toward negative scores and PDO samples toward positive scores, indicating a strong compositional gradient between the PDO and PGI sample groups. The QC samples were positioned between the PDO and PGI clusters, reflecting their composite nature, derived from pooled aliquots of all study samples, and supporting the representativeness of the QC injections.

The corresponding loading plot (Figure 3B) shows that most of the identified features contributing are aligned along PC1. Furanoids, esters, lactones, aldehydes, acids, phenols, and alcohols displayed predominantly positive loadings on PC1, whereas terpenoids were largely associated with negative PC1 loadings. Interestingly, most significant features exhibited positive PC1 loadings and higher relative abundances in PDO samples, whereas only a small subset of variables (mainly terpenoids) showed negative PC1 loadings and were enriched in PGI vinegars. This unbalanced distribution of discriminant volatiles toward the PDO side suggests that, within the present dataset, the PDO samples exhibit a comparatively richer volatile signature, consistent with their longer maturation and more extensive production process.

**FIGURE 4:**
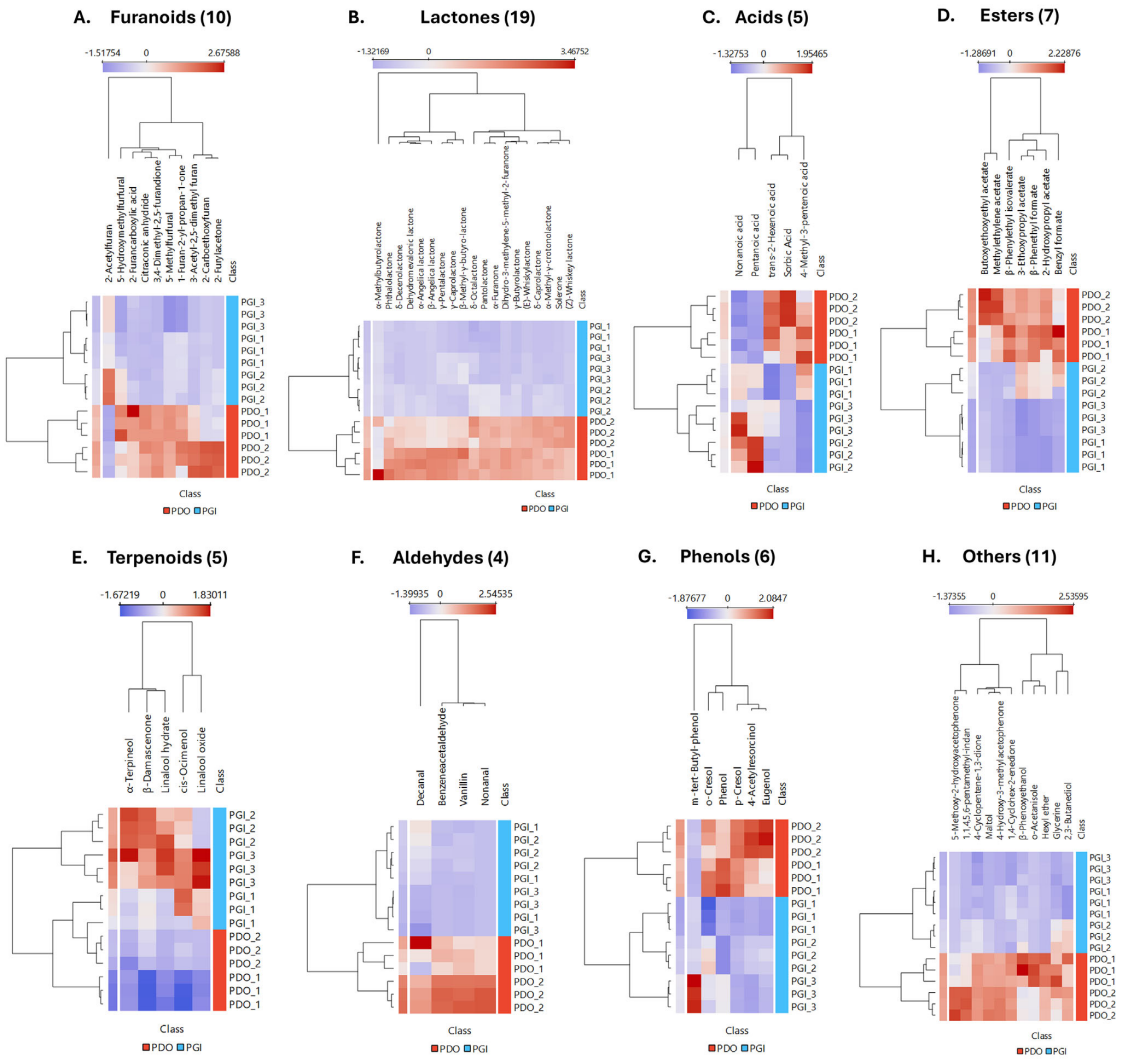
Hierarchical cluster analysis (HCA) heatmaps for each chemical class of compounds in Balsamic vinegar (BV), based on normalized peak areas of putatively identified significant compounds. Samples (rows) and compounds (columns) are clustered according to Euclidean distance and Ward's linkage. Color scale represents relative abundance (blue = low, red = high).

To further elucidate the chemical patterns underlying the separation observed in the PCA, HCA was performed independently on the main chemical class (Figure 4), revealing clear differences between PDO and PGI samples. While PCA summarizes the global structure of the volatilomic dataset, class‐specific heatmaps enable a more detailed examination of how individual compound groups contribute to this separation. For each class, the HCA clearly partitioned PDO and PGI samples into distinct clusters, consistent with the PCA score‐plot distribution, revealing characteristic intensity trends within each chemical family and providing a granular chemical context for PDO‐PGI sample differentiation.

Furanoid compounds showed higher relative abundances in the PDO samples analyzed (Figure 4A), emerging as one of the chemical classes most associated with the PDO‐PGI contrast within the present dataset and aligning with technological differences in their processing. Furans originate predominantly from thermal degradation of carbohydrates, including pathways associated with caramelization and Maillard‐type reactions, both of which are intensified during the prolonged cooking of grape must in PDO production. Antonelli et al. described that the must concentration for traditional balsamic vinegar (PDO) involves heating at 80‐90°C for many hours, a process that promotes the transformation of sugars into furan derivatives [1]. Similarly, Chinnici et al. report that the cooking phase of PDO vinegars favors the formation of a wide range of Maillard‐ and caramelization‐derived volatiles, whereas this step is minimal in PGI production [2].

Beyond must cooking, the long‐term aging characteristic of PDO vinegars further amplifies the accumulation of furanoid species. Oxidative conditions, water loss, and sugar transformations during the multiyear maturation period are known to favor their concentration, a trend also observed in cooked‐must vinegars and long‐aged wine vinegars, where furanoids are recognized as indicators of both thermal intensity and extended aging [14, 18].

Based on literature‐reported odor descriptors, the putatively identified furanoids are commonly associated with sweet and caramel‐like notes. Several of the furanoid compounds identified in the present dataset, including 2‐acetylfuran, 5‐methylfurfural, 5‐hydroxymethylfurfural, and related derivatives, have been reported as markers of heat processing and aging in BV [2, 6, 14, 19, 20, 21, 22, 23, 24, 25].

Lactones also exhibited higher abundances in the PDO samples analyzed (Figure 4B), which is consistent with previous studies on BV and wine vinegars, which report lactones as characteristic products of fermentation‐related reactions, including hydroxyacid cyclization and sugar degradation, as well as extraction during wood aging. Such processes are expected to be more extensive in long‐aged PDO products than in PGI vinegars, where the cooked‐must fraction is considerably smaller, and the product matures for a much shorter period, generally in a single oak vessel [2, 26].

According to reported odor descriptors in the literature, the lactones identified in the present dataset are commonly associated with sweet, caramel‐like, and creamy‐coconut nuances. For instance, α‐ and β‐angelica lactone, α‐furanone, and several methyl‐substituted lactones have been described as exhibiting sweet and caramellic characteristics. Medium‐chain γ‐ and δ‐lactones, such as γ‐ and δ‐caprolactone, and δ‐octalactone, are frequently reported as contributing coconut‐ and creamy‐notes. Moreover, whiskey lactones, well‐known oak‐derived compounds, are commonly associated with coconut, woody, toasted, and sweet notes in barrel‐aged wines and spirits [1, 2, 3, 9, 14, 18, 19, 21, 26, 27, 28].

Most volatile acids (Figure 4C) also exhibited higher relative abundances in the PDO samples, consistent with previous studies reporting that short‐ and medium‐chain acids tend to accumulate during vinegar maturation and may contribute to the differentiation between production systems and aging regimes [18, 26].

A similar trend was observed for esters (Figure 4D), which tended to have higher relative abundances in the PDO samples analyzed, which is consistent with previous studies on balsamic and wine vinegars reporting that esters tend to be more abundant in long‐aged vinegars, reflecting ongoing reactions between organic acids and alcohols under acidic conditions and in contact with wood. Several esters putatively identified in this study are reported in the literature to impart sweet, fruity, and floral notes, such as methylethylene acetate, benzyl formate, β‐phenethyl formate, and β‐phenethyl isovalerate [2, 6, 18, 19, 21, 26].

In contrast to the other classes, most identified terpenoids showed higher relative abundances in the analyzed PGI samples than in the PDO samples (Figure 4E), which is consistent with their closer association with grape‐derived, varietal volatile composition. Studies in wine have reported that several monoterpenoids and other grape‐derived volatiles tend to decrease or undergo transformation during aging, with the concurrent build‐up of oxidation and cyclization products associated with more mature aroma profiles [29, 30, 31]. In this context, the elevated terpenoid levels observed in the PGI samples may indicate partial preservation of fresher, grape‐derived signatures, consistent with their shorter, less intensive maturation processes [2, 3, 9, 19, 21, 26, 32, 33].

Most identified aldehydes displayed higher relative responses in PDO than in PGI samples (Figure 4F). Volatile aldehydes in vinegars arise mainly from ethanol oxidation and from extraction during prolonged contact with wooden barrels, and tend to be more abundant in products subjected to long‐term traditional aging in wood [6, 26]. Among the aldehydes identified in the dataset, benzeneacetaldehyde has been associated in literature with green, sweet, and floral notes, whereas nonanal is commonly described as waxy, fresh, and fatty, and decanal as sweet, waxy, and citrus‐like. Vanillin, a classic oak‐derived aldehyde, is widely associated with sweet, vanilla‐like notes [3, 6, 18, 19, 26, 32, 33].

Phenolic volatiles also differed between the PDO and PGI samples analyzed (Figure 4G), with most phenolic compounds showing higher relative intensities in PDO samples, consistent with their known association with wood contact and long‐term aging. Phenol and the cresols (*o*‐ and *p*‐cresol) have been described in the literature as being associated with “phenolic”, smoky, and medicinal aroma. These compounds arise predominantly from direct extraction and from thermal‐induced transformations of lignin‐derived structures, and have been reported to increase during vinegar aging in wood [18, 19, 22, 23, 28, 33, 34, 35, 36].

Beyond the major chemical classes, a small group of compounds categorized as “Other” also contributed to the differentiation between PDO and PGI products analyzed (Figure 4H). Most of these features exhibited higher relative abundances in PDO samples, consistent with the overall trend observed in the dataset. Among them, maltol has been described in the literature as associated with sweet, caramellic, and fruity nuances and with sugar degradation pathways and heat‐induced transformations in cooked musts, aligning with the more intensive thermal processing and extended maturation associated with PDO vinegar production [37, 38].

Overall, the multivariate analyses revealed a coherent, internally consistent structure in the volatilomic dataset, separating the PDO and PGI products analyzed. The consistency observed across global and class‐specific analyses supports the workflow's analytical robustness and reinforces the chemical interpretability of the detected trends. While these findings apply to the samples investigated, they illustrate how comprehensive volatilomic profiling can translate technological variables into structured chemical signatures within complex food matrices.

## 4 Conclusions

This study demonstrates that HS‐SPME coupled to GC×GC‐TOFMS is a suitable analytical tool for resolving and characterizing the volatilomic profiles of balsamic vinegars, and for exploring the chemical differentiation of the selected Protected Designation of Origin (PDO) and Protected Geographical Indication (PGI) products. Comparative evaluation of extraction conditions, particularly within the use of a DVB/CAR/PDMS fiber combined with NaCl‐assisted headspace extraction, enabled broad chemical coverage and satisfactory analytical repeatability.

Through a structured feature‐selection workflow, discriminant volatiles were identified across multiple chemical classes. Univariate and multivariate analyses converged on a consistent chemical pattern: within the present dataset, PDO vinegars exhibited higher relative abundances of furanoids, lactones, esters, aldehydes, acids, and phenols, compound classes commonly reported in the literature as associated with must cooking, long‐term maturation, and wood‐mediated reactions. On the other hand, PGI vinegars showed comparatively higher levels of grape‐derived terpenoids, consistent with their shorter, less transformative aging regime. Based on reported odor descriptors in the literature, these compositional trends may be associated with sweeter, caramel‐like, creamy, balsamic, woody, and floral nuances in PDO samples, and with fresher citrus‐floral varietal notes in PGI samples.

Overall, volatilomic profiling using HS‐SPME‐GC×GC‐TOFMS revealed structured compositional differences among the analyzed products, consistent with the distinct production approaches. Although the number of samples limits generalization across denominations, the study establishes a methodological foundation for future investigations incorporating larger datasets, longitudinal aging studies, and sensory validation, thereby consolidating its applicability to authenticity assessment and quality control.

## Author Contributions

**Flavio A. Franchina**: methodology, investigation, data curation, writing – review and editing, supervision, resources. **Alberto Cavazzini**: resources. **Monica Romagnoli**: methodology, writing – review and editing, and formal analysis. **Allan Polidoro**: writing – original draft, writing – review and editing, visualization, software, and data curation. **Sofia Malcangi**: investigation, data curation, formal analysis, and writing – review and editing.

## Conflicts of Interest

The authors declare no conflicts of interest.

## Declarations

During the preparation of this manuscript, the authors used grammar checker tools to refine the English language and syntax.

## Supporting information




**Supporting File 1**: jssc70442‐sup‐0001‐SuppMat.docx.

**Supporting File 2**: jssc70442‐sup‐0002‐FigureS1.tif.

**Supporting File 3**: jssc70442‐sup‐0003‐FigureS2.tif.

**Supporting File 4**: jssc70442‐sup‐0004‐FigureS3.tif.

## Data Availability

Data available on request from the authors.
