# Supplementary material for: Volatilomic Differentiation of Protected‐Origin Italian Balsamic Vinegars by HS‐SPME‐GC×GC‐TOFMS
Source: J Sep Sci. 2026 May 11;49:e70442. doi: 10.1002/jssc.70442 (PMC13159424; doi:10.1002/jssc.70442)
Supplement: Supplementary file 1 — Supporting File 1: jssc70442‐sup‐0001‐SuppMat.docx. [file JSSC-49-e70442-s004.docx]

**SUPPLEMENTARY MATERIAL**

**Volatilomic Differentiation of Protected-Origin Italian Balsamic Vinegars by HS-SPME-GC×GC-TOFMS**

Sofia Malcangi^1,#^, Allan Polidoro^2,^^#^, Monica Romagnoli^1^, Alberto Cavazzini^1^, Flavio A. Franchina^1,^*.

^1^ Department of Chemical, Pharmaceutical, and Agricultural Sciences, University of Ferrara, Via L. Borsari 46, 44121 Ferrara, Italy

^2^ Department of Environmental and Prevention Sciences, University of Ferrara, Via L. Borsari 46, 44121, Ferrara, Italy

^#^These authors contributed equally to this work

*Corresponding author: Flavio A. Franchina, PhD - Phone: +39 (0532) 455836 - E-mail address: frnfvn@unife.it, flaviofranchina@gmail.com

Table of Contents

[Figure S1 2](#_Toc227568917)

[Section S1. Evaluation of the HS-SPME methodology 3](#_Toc227568918)

[Selection of fiber coating 3](#_Toc227568919)

[Figure S2. 4](#_Toc227568920)

[Sodium chloride addition 5](#_Toc227568921)

[Figure S3. 6](#_Toc227568922)

[Table S1. 7](#_Toc227568923)

[References 10](#_Toc227568924)


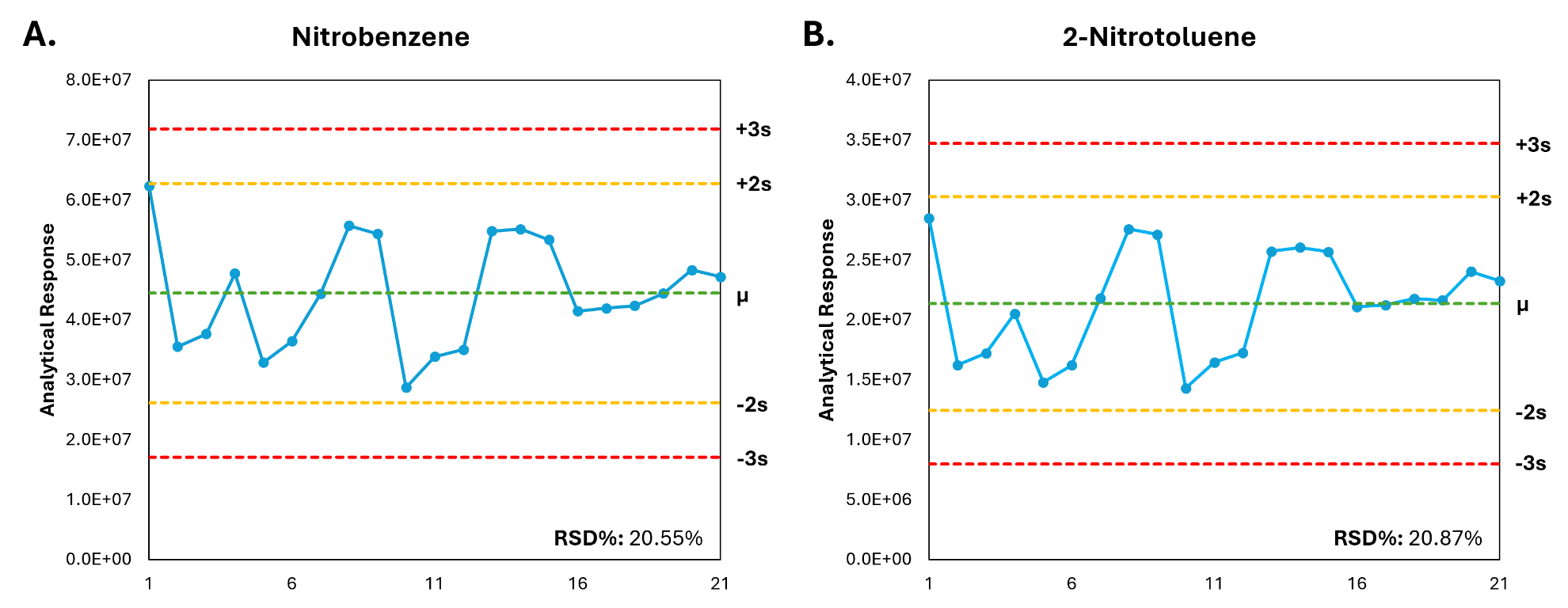


Figure S1**. C**ontrol charts for the analytical response of (A) nitrobenzene and (B) 2-nitrotoluene measured across the analytical sequence. The central dashed line represents the mean response (μ), while the dashed lines at ±2s and ±3s indicate the warning and control limits, respectively, used to assess analytical stability and reproducibility over time.

**Section S1. Evaluation of the HS-SPME methodology**

Evaluating the HS-SPME conditions is important to ensure reliable and comprehensive characterization of the volatile fraction of BV. Since this matrix contains chemically diverse compounds, preliminary experiments were conducted to evaluate extraction parameters that improve analytical response and provide suitable volatilomic coverage [1,2].

**Selection of fiber coating**

The choice of SPME fiber coating is a fundamental factor in evaluating volatile extraction efficiency, as it determines the chemical coverage and the representativeness of the resulting volatile profile [1]. In this study, the PDMS/DVB and DVB/CAR/PDMS coatings were evaluated to comparatively assess extraction efficiency and chemical coverage of compounds with diverse volatilities and polarities, typically found in BVs [2–4]. All tests were conducted using HS-SPME-GC×GC-TOFMS, with a pooled QC sample prepared by combining aliquots of all samples under investigation to represent the overall chemical composition of the dataset. The comparison between the two coatings with respect to total analytical response and the relative contributions of different chemical classes is shown in **Figure S2**.


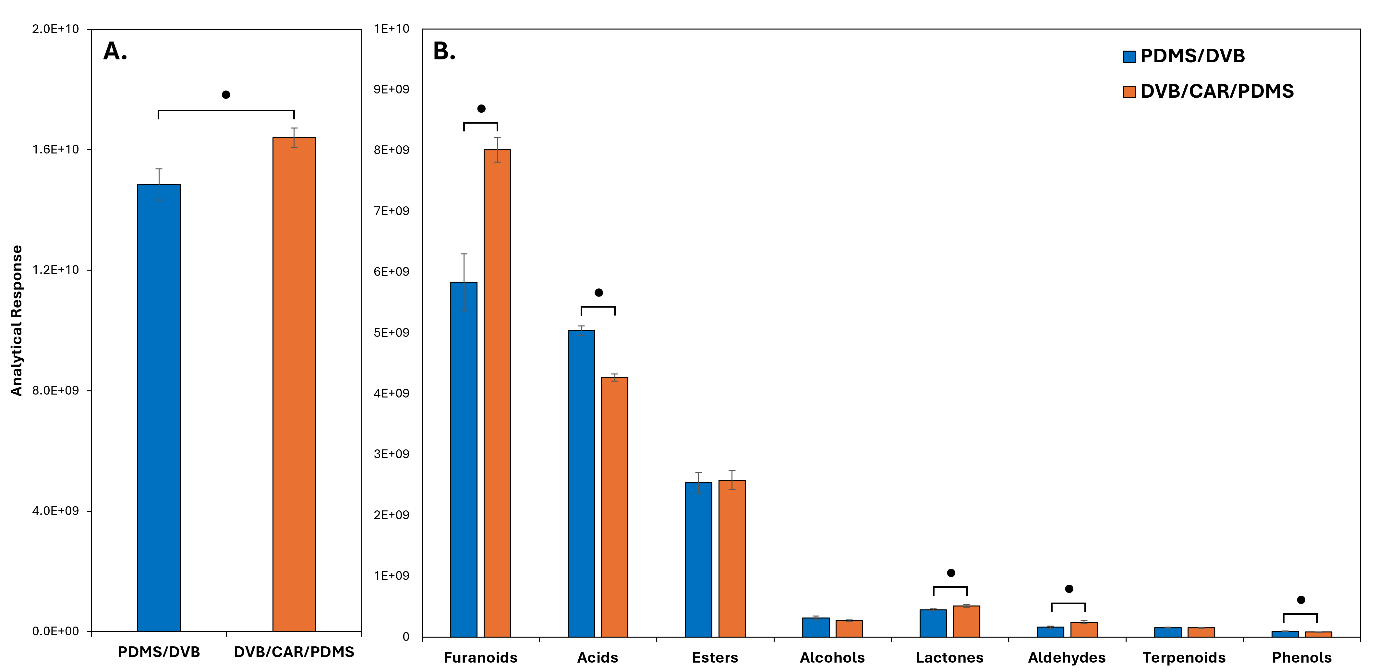


Figure S2. Comparison of (A) total analytical response (TIC) and (B) distribution of chemical class responses obtained using PDMS/DVB and DVB/CAR/PDMS fibers for the HS-SPME-GC×GC-TOFMS analysis of BV volatiles. Error bars represent standard deviations (*n* = 3), and statistically significant differences (p < 0.05) are indicated by “•”.

The comparison between the fiber coatings revealed significant differences in extraction performance. Assessing the total analytical response (**Figure S2A**), the DVB/CAR/PDMS coating produced a statistically significant (p < 0.05) higher analytical response than PDMS/DVB, indicating greater efficiency in extracting the volatile constituents of BV. This enhanced performance was evident not only in the cumulative signal but also across several chemical classes (**Figure S2B**), for which DVB/CAR/PDMS yielded higher responses. These results are in agreement with previous studies on similar matrices, which reported broader analytical coverage for this triphasic coating [1,5]. Namely, furanoids, lactones, and aldehydes presented higher relative responses under the tested conditions, suggesting that the multilayer structure of this coating enhances the extraction of compounds with diverse physicochemical properties. This synergistic combination results in a broader sorptive range, allowing DVB/CAR/PDMS to extract structurally varied compounds from the complex BV matrix more effectively.

Although a slightly higher response for organic acids and phenolic compounds was observed with PDMS/DVB, no statistically significant differences were observed for esters and terpenoids between the two fibers, indicating comparable extraction efficiency for these classes under the tested conditions.

Importantly, in addition to producing higher recoveries for most classes, DVB/CAR/PDMS demonstrated higher repeatability, with lower RSD% values for most compound classes compared with PDMS/DVB, a behavior also reported in previous studies [1,6,7].

Therefore, under the conditions evaluated, the DVB/CAR/PDMS coating provided the highest overall analytical response while maintaining consistent performance across most compound classes. Considering the chemical diversity of the BV matrix and the need for comprehensive volatilomic characterization, the DVB/CAR/PDMS coating was selected for all subsequent analyses.

**Sodium chloride addition**

The ionic strength of the sample matrix is another important parameter that influences extraction efficiency in HS-SPME, especially in aqueous food systems such as wines and vinegars. The addition of inorganic salts is known to promote the transfer of volatile organic compounds from the liquid phase to the HS by reducing their solubility, a phenomenon described as the salting-out effect [8–11]. Because BV contains a diverse array of oxygenated and semi-volatile compounds with varying polarities, assessing the impact of salt addition is important to evaluate its effect on analytical response and volatilome coverage. In this study, the influence of a high ionic-strength condition (40% w/v NaCl) on HS-SPME extraction performance was evaluated using the pooled QC sample. **Figure S3** presents the comparison between salted and non-salted extractions in terms of total analytical response (**Figure S3A**) and the distribution of chemical class responses (**Figure S3B**).


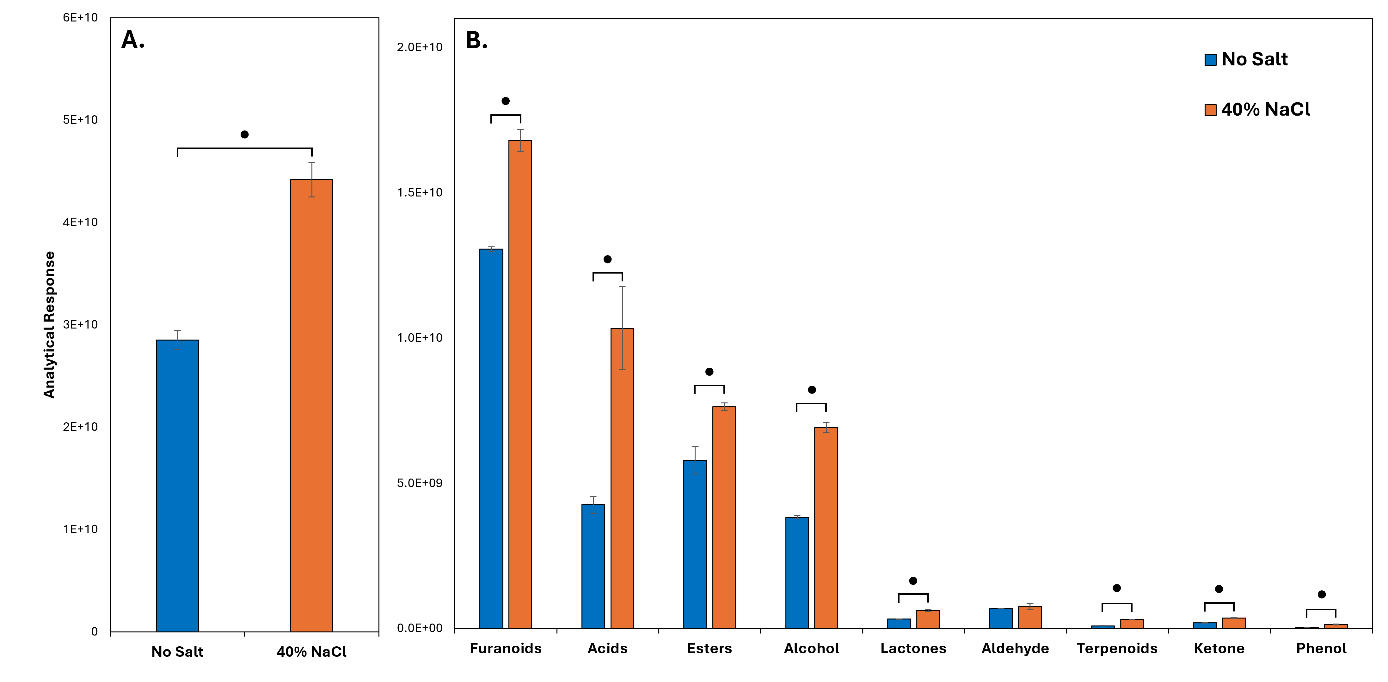


Figure S3. Comparison of (A) total analytical response and (B) distribution of chemical class responses obtained for the HS-SPME of BV volatiles with and without NaCl addition (40% w/v). Error bars represent standard deviations (*n* = 3), and statistically significant differences (p < 0.05) are indicated by “•”.

The addition of NaCl significantly increased the overall analytical response of BV volatiles. As shown in **Figure S3A**, the total analytical response increased significantly (*p* < 0.05) when NaCl was added at 40% (*w/v*), confirming the expected salting-out effect. Increasing the ionic strength reduces the solubility of organic compounds in the aqueous phase, favoring their transfer to the headspace and enhancing their partitioning into the SPME coating. This behavior is consistent with previous observations in aqueous food matrices, including wines and vinegars [8–10].

At the level of chemical classes (**Figure S3B**), the extraction enhancement was not uniform but followed a consistent general trend. Furanoids, esters, alcohols, acids, lactones, terpenoids, and phenols showed significantly higher responses in salted extractions, thereby enhancing the relative response of compounds spanning a wide range of volatilities and polarities, without narrowing chemical coverage.

Therefore, under the tested conditions, the addition of 40% (w/v) NaCl improved the overall analytical response across diverse chemical classes. Given the consistent analytical benefits observed under this comparative evaluation, NaCl addition was adopted for all subsequent analyses.

Table S1. Annotated features significant after Benjamini–Hochberg FDR correction (Mann-Whitney U test; FDR-adjusted p < 0.05) detected by HS-SPME-GC×GC-TOFMS that discriminate PDO and PGI samples, identified under the *Putative ID^i^* and *Putative ID^ii^* criteria.

| **Compound** | **CAS** | **Chem.**  **Class** | **Aroma descriptors** | **^1^t_R_**  **(min)** | **^2^t_R_**  **(s)** | **Match**  **(‰)** | **RI_Exp_** | **RI_Lib_** | **Characteristic**  **m/z** |
| --- | --- | --- | --- | --- | --- | --- | --- | --- | --- |
| 2,3-Butanediol | 513-85-9 | Other | Fruity, creamy | 3.95 | 2.01 | 951 | 799 | 787 | 57 |
| α-Angelica lactone | 591-12-8 | Lactone | Sweet | 5.55 | 2.60 | 930 | 872 | 871 | 97 |
| Pentanoic acid | 109-52-4 | Acid | Cheesy, acidic | 5.80 | 1.84 | 845 | 882 | 901 | 73 |
| 2-Hydroxypropyl acetate | 627-69-0 | Ester | - | 5.85 | 2.30 | 904 | 885 | 883 | 74 |
| 4-Cyclopentene-1,3-dione | 930-60-9 | Other | - | 5.90 | 2.99 | 908 | 888 | 883 | 96 |
| 2-Acetylfuran | 1192-62-7 | Furanoid | Sweet, caramellic | 6.50 | 2.54 | 946 | 914 | 911 | 95 |
| γ-Butyrolactone | 96-48-0 | Lactone | Caramellic | 6.50 | 0.57 | 967 | 913 | 915 | 86 |
| α-Furanone | 20825-71-2 | Lactone | Caramellic | 6.55 | 0.57 | 806 | 915 | 914 | 84 |
| β-Angelica lactone | 591-11-7 | Lactone | Caramellic | 7.15 | 0.06 | 971 | 942 | 945 | 55 |
| Citraconic anhydride | 616-02-4 | Furanoid | - | 7.20 | 0.05 | 844 | 944 | 949 | 68 |
| α-Methylbutyrolactone | 1679-47-6 | Lactone | Caramellic | 7.35 | 0.15 | 782 | 951 | 953 | 100 |
| 2-Furylacetone | 6975-60-6 | Furanoid | Caramellic | 7.40 | 2.49 | 853 | 955 | 952 | 124 |
| γ-Pentalactone | 108-29-2 | Lactone | Sweet | 7.45 | 0.19 | 959 | 955 | 956 | 85 |
| β-Methyl-γ-butyro-lactone | 1679-49-8 | Lactone | - | 7.55 | 0.31 | 794 | 960 | 960 | 100 |
| 5-Methylfurfural | 620-02-0 | Furanoid | Sweet, caramellic | 7.60 | 2.83 | 945 | 964 | 964 | 110 |
| Glycerine | 56-81-5 | Alcohol | - | 7.65 | 2.98 | 952 | 966 | 950 | 61 |
| α-Methyl-γ-crotonolactone | 22122-36-7 | Lactone | - | 8.00 | 0.56 | 863 | 980 | 983 | 98 |
| Phenol | 108-95-2 | Phenol | Phenolic | 8.00 | 2.51 | 946 | 982 | 981 | 94 |
| 4-Methyl-3-pentenoic acid | 504-85-8 | Acid | - | 8.15 | 0.09 | 884 | 987 | 989 | 99 |
| 3-Ethoxypropyl acetate | - | Ester | - | 8.40 | 2.07 | 813 | 999 | 1019 | 86 |
| 1-Furan-2-yl-propan-1-one | 3194-15-8 | Furanoid | - | 8.65 | 2.52 | 930 | 1011 | 1012 | 95 |
| *trans*-2-Hexenoic acid | 13419-69-7 | Acid | Fruity, sweet | 8.70 | 1.99 | 860 | 1013 | 1005 | 99 |
| Methylethylene acetate | 623-84-7 | Ester | Fruity | 9.10 | 2.50 | 901 | 1031 | 1019 | 100 |
| 1,4-Cyclohex-2-enedione | 4505-38-8 | Other | - | 9.10 | 2.96 | 855 | 1032 | 1032 | 110 |
| Pantolactone | 79-50-5 | Lactone | - | 9.20 | 2.98 | 925 | 1036 | 1032 | 71 |
| 3,4-Dimethyl-2,5-furandione | 766-39-2 | Furanoid | - | 9.35 | 0.06 | 969 | 1041 | 1038 | 126 |
| Benzeneacetaldehyde | 122-78-1 | Aldehyde | Green, sweet, floral | 9.45 | 2.84 | 960 | 1047 | 1045 | 99 |
| 2-Carboethoxyfuran | 614-99-3 | Furanoid | Fruity | 9.60 | 2.68 | 787 | 1054 | 1047 | 95 |
| *o*-Cresol | 95-48-7 | Phenol | Phenolic | 9.65 | 2.55 | 817 | 1056 | 1054 | 108 |
| γ‐Caprolactone | 695-06-7 | Lactone | Coconut, sweet | 9.70 | 0.29 | 959 | 1057 | 1056 | 85 |
| Sorbic Acid | 110-44-1 | Acid | - | 9.95 | 2.88 | 861 | 1070 | 1060 | 112 |
| *p*-Cresol | 106-44-5 | Phenol | Phenolic | 10.11 | 2.58 | 932 | 1076 | 1077 | 107 |
| Linalool oxide | 5989-33-3 | Terpenoid | Earthy, floral | 10.11 | 2.04 | 888 | 1076 | 1074 | 111 |
| Benzyl formate | 104-57-4 | Ester | Floral, fruity, spicy | 10.16 | 2.76 | 882 | 1079 | 1079 | 136 |
| Dihydro-3-methylene-5-methyl-2-furanone | 62873-16-9 | Lactone | - | 10.16 | 0.70 | 870 | 1077 | 1075 | 68 |
| 3-Acetyl-2,5-dimethyl furan | 10599-70-9 | Furanoid | Sweet, nutty | 10.31 | 2.54 | 914 | 1085 | 1095 | 138 |
| Nonanal | 124-19-6 | Aldehyde | Waxy, fresh, fatty | 10.46 | 2.44 | 946 | 1092 | 1104 | 98 |
| 2-Furancarboxylic acid | 88-14-2 | Furanoid | - | 10.56 | 2.30 | 773 | 1096 | 1092 | 112 |
| δ-Caprolactone | 823-22-3 | Lactone | Creamy, fruity, coconut | 10.56 | 0.49 | 933 | 1095 | 1095 | 70 |
| Maltol | 118-71-8 | Other | Sweet, caramellic, fruity | 10.96 | 0.01 | 896 | 1113 | 1110 | 126 |
| Solerone | 29393-32-6 | Lactone | - | 11.26 | 0.95 | 973 | 1128 | 1134 | 85 |
| *cis*-Ocimenol | 7643-59-6 | Terpenoid | - | 11.76 | 2.15 | 879 | 1153 | 1155 | 136 |
| Dehydromevalonic lactone | 2381-87-5 | Lactone | - | 12.06 | 1.03 | 944 | 1166 | 1169 | 112 |
| β-Phenethyl formate | 104-62-1 | Ester | Floral, green | 12.31 | 2.76 | 967 | 1179 | 1178 | 104 |
| α-Terpineol | 98-55-5 | Terpenoid | Citrus, floral, pine | 12.66 | 2.24 | 915 | 1195 | 1189 | 136 |
| Decanal | 112-31-2 | Aldehyde | Sweet, waxy, citrus | 12.91 | 1.97 | 937 | 1207 | 1206 | 112 |
| β-Phenoxyethanol | 122-99-6 | Alcohol | Balsam, rose | 13.21 | 2.98 | 828 | 1223 | 1225 | 94 |
| 5-Hydroxymethylfurfural | 67-47-0 | Furanoid | Caramellic, Buttery | 13.31 | 0.61 | 903 | 1226 | 1232 | 126 |
| Linalool hydrate | 29210-77-3 | Terpenoid | - | 13.36 | 2.30 | 799 | 1230 | 1237 | 71 |
| Hexyl ether | 112-58-3 | Other | - | 13.96 | 2.28 | 793 | 1259 | 1264 | 85 |
| Nonanoic acid | 112-05-0 | Acid | Cheesy, waxy | 14.16 | 2.02 | 933 | 1269 | 1273 | 129 |
| o-Acetanisole | 579-74-8 | Other | Anisic, almond | 14.31 | 2.83 | 770 | 1277 | 1290 | 135 |
| 4-Acetylresorcinol | 89-84-9 | Phenol | - | 14.41 | 0.02 | 769 | 1280 | 1276 | 137 |
| δ-Octalactone | 698-76-0 | Lactone | Coconut, sweet, fatty | 14.61 | 0.18 | 819 | 1290 | 1287 | 99 |
| (*E*)-Whiskylactone | 39638-67-0 | Lactone | Coconut, spicy | 14.66 | 2.73 | 941 | 1295 | 1288 | 99 |
| *m*-tert-Butyl-phenol | 585-34-2 | Phenol | - | 14.66 | 2.52 | 822 | 1294 | 1296 | 135 |
| 4-Hydroxy-3-methylacetophenone | 876-02-8 | Other | - | 14.91 | 2.86 | 872 | 1308 | 1323 | 135 |
| (*Z*)-Whiskey lactone | 55013-32-6 | Lactone | Sweet, spicy, coconut | 15.26 | 2.93 | 921 | 1326 | 1337 | 99 |
| Phthalolactone | 87-41-2 | Lactone | Sweet, coconut | 15.81 | 1.24 | 874 | 1354 | 1351 | 134 |
| Eugenol | 97-53-0 | Phenol | Sweet, spicy, woody | 15.96 | 2.80 | 834 | 1363 | 1357 | 164 |
| Butoxyethoxyethyl acetate | 124-17-4 | Ester | - | 16.06 | 2.40 | 900 | 1368 | 1366 | 87 |
| β-Damascenone | 23726-93-4 | Terpenoid | Apple, floral, honey | 16.46 | 2.46 | 875 | 1389 | 1386 | 121 |
| Vanillin | 121-33-5 | Aldehyde | Sweet, vanilla | 16.76 | 0.53 | 859 | 1403 | 1404 | 152 |
| 5-Methoxy-2-hydroxyacetophenone | 705-15-7 | Other | - | 17.36 | 0.26 | 760 | 1436 | 1442 | 151 |
| δ-Decenolactone | 54814-64-1 | Lactone | Sweet, creamy, coconut | 18.21 | 0.12 | 877 | 1484 | 1501 | 97 |
| β-Phenylethyl isovalerate | 140-26-1 | Ester | Fruity, floral, sweet | 18.41 | 2.52 | 896 | 1497 | 1490 | 104 |
| 1,1,4,5,6-pentamethyl-indan | 16204-67-4 | Other | - | 18.76 | 2.88 | 773 | 1518 | 1522 | 173 |

RI_𝐸𝑥𝑝_: experimental linear retention index calculated using a C_7_-C_30_ alkane series.

RI_𝐿𝑖𝑏_: reference retention index from the NIST23 library.

p-adj BH: p-values adjusted for multiple comparisons using the Benjamini-Hochberg false discovery rate (FDR)

Aroma descriptors from literature [12–19].

# References

[1] Paula Barros, E., Moreira, N., Elias Pereira, G., Leite, S. G. F., Moraes Rezende, C., Guedes De Pinho, P., Development and validation of automatic HS-SPME with a gas chromatography-ion trap/mass spectrometry method for analysis of volatiles in wines. *Talanta* 2012, 101, 177–186.

[2] Marrufo-Curtido, A., Cejudo-Bastante, M. J., Durán-Guerrero, E., Castro-Mejías, R., Natera-Marín, R., Chinnici, F., García-Barroso, C., Characterization and differentiation of high quality vinegars by stir bar sorptive extraction coupled to gas chromatography-mass spectrometry (SBSE–GC–MS). *LWT* 2012, 47, 332–341.

[3] Chinnici, F., Durán Guerrero, E., Sonni, F., Natali, N., Natera Marín, R., Riponi, C., Gas Chromatography−Mass Spectrometry (GC−MS) Characterization of Volatile Compounds in Quality Vinegars with Protected European Geographical Indication. *J. Agric. Food Chem.* 2009, 57, 4784–4792.

[4] Callejón, R. M., Torija, M. J., Mas, A., Morales, M. L., Troncoso, A. M., Changes of volatile compounds in wine vinegars during their elaboration in barrels made from different woods. *Food Chemistry* 2010, 120, 561–571.

[5] Rossi, L., Foschi, M., Biancolillo, A., Maggi, M. A., D’Archivio, A. A., Optimization of HS-SPME-GC/MS Analysis of Wine Volatiles Supported by Chemometrics for the Aroma Profiling of Trebbiano d’Abruzzo and Pecorino White Wines Produced in Abruzzo (Italy). *Molecules* 2023, 28, 1534.

[6] Pizarro, C., Pérez‐del‐Notario, N., González‐Sáiz, J. M., Headspace solid‐phase microextraction for direct determination of volatile phenols in cider. *J of Separation Science* 2009, 32, 3746–3754.

[7] Rios, J. J., Morales, A., Márquez-Ruiz, G., Headspace solid-phase microextraction of oil matrices heated at high temperature and phthalate esters determination by gas chromatography multistage mass spectrometry. *Talanta* 2010, 80, 2076–2082.

[8] Rocha, S., Ramalheira, V., Barros, A., Delgadillo, I., Coimbra, M. A., Headspace Solid Phase Microextraction (SPME) Analysis of Flavor Compounds in Wines. Effect of the Matrix Volatile Composition in the Relative Response Factors in a Wine Model. *J. Agric. Food Chem.* 2001, 49, 5142–5151.

[9] Yu, Y.-J., Lu, Z.-M., Yu, N.-H., Xu, W., Li, G.-Q., Shi, J.-S., Xu, Z.-H., HS-SPME/GC-MS and chemometrics for volatile composition of Chinese traditional aromatic vinegar in the Zhenjiang region: Volatile composition of Zhenjiang aromatic vinegar. *J. Inst. Brew.* 2012, 118, 133–141.

[10] Zhu, H., Zhu, J., Wang, L., Li, Z., Development of a SPME-GC-MS method for the determination of volatile compounds in Shanxi aged vinegar and its analytical characterization by aroma wheel. *J Food Sci Technol* 2016, 53, 171–183.

[11] Rodríguez‐Bencomo, J. J., Muñoz‐González, C., Andújar‐Ortiz, I., Martín‐Álvarez, P. J., Moreno‐Arribas, M. V., Pozo‐Bayón, M. Á., Assessment of the effect of the non‐volatile wine matrix on the volatility of typical wine aroma compounds by headspace solid phase microextraction/gas chromatography analysis. *J Sci Food Agric* 2011, 91, 2484–2494.

[12] The Good Scents Company, The Good Scents Company - Search for product information, https://www.thegoodscentscompany.com/search.html (last time accessed: November 18, 2025).

[13] Bordiga, M., Nollet, L. M. L., Food Aroma Evolution: During Food Processing, Cooking, and Aging. CRC Press, 1st Edition. | Boca Raton : CRC Press, 2019. | Series: Food Analysis & Properties, 2475-7551 2019.

[14] Li, N., Li, G., Guan, X., Li, A., Tao, Y., Volatile aroma compound-based decoding and prediction of sweet berry aromas in dry red wine. *Food Chemistry* 2025, 463, 141248.

[15] Sánchez-Palomo, E., Trujillo, M., García Ruiz, A., González Viñas, M. A., Aroma profile of malbec red wines from La Mancha region: Chemical and sensory characterization. *Food Research International* 2017, 100, 201–208.

[16] Yan, X., Pan, S., Liu, X., Tan, M., Zheng, X., Du, W., Wu, M., Song, Y., Profiling the Major Aroma-Active Compounds of Microwave-Dried Jujube Slices through Molecular Sensory Science Approaches. *Foods* 2023, 12, 3012.

[17] Bento-Silva, A., Duarte, N., Santos, M., Costa, C. P., Vaz Patto, M. C., Rocha, S. M., Bronze, M. R., Comprehensive Two-Dimensional Gas Chromatography as a Powerful Strategy for the Exploration of Broas Volatile Composition. *Molecules* 2022, 27, 2728.

[18] Dunkel, A., Steinhaus, M., Kotthoff, M., Nowak, B., Krautwurst, D., Schieberle, P., Hofmann, T., Nature’s Chemical Signatures in Human Olfaction: A Foodborne Perspective for Future Biotechnology. *Angew Chem Int Ed* 2014, 53, 7124–7143.

[19] Durán-Guerrero, E., Castro, R., García-Moreno, M. D. V., Rodríguez-Dodero, M. D. C., Schwarz, M., Guillén-Sánchez, D., Aroma of Sherry Products: A Review. *Foods* 2021, 10, 753.
