# Supplementary figures and images for: Volatilomic Differentiation of Protected‐Origin Italian Balsamic Vinegars by HS‐SPME‐GC×GC‐TOFMS
Source: J Sep Sci. 2026 May 11;49:e70442. doi: 10.1002/jssc.70442 (PMC13159424; doi:10.1002/jssc.70442)

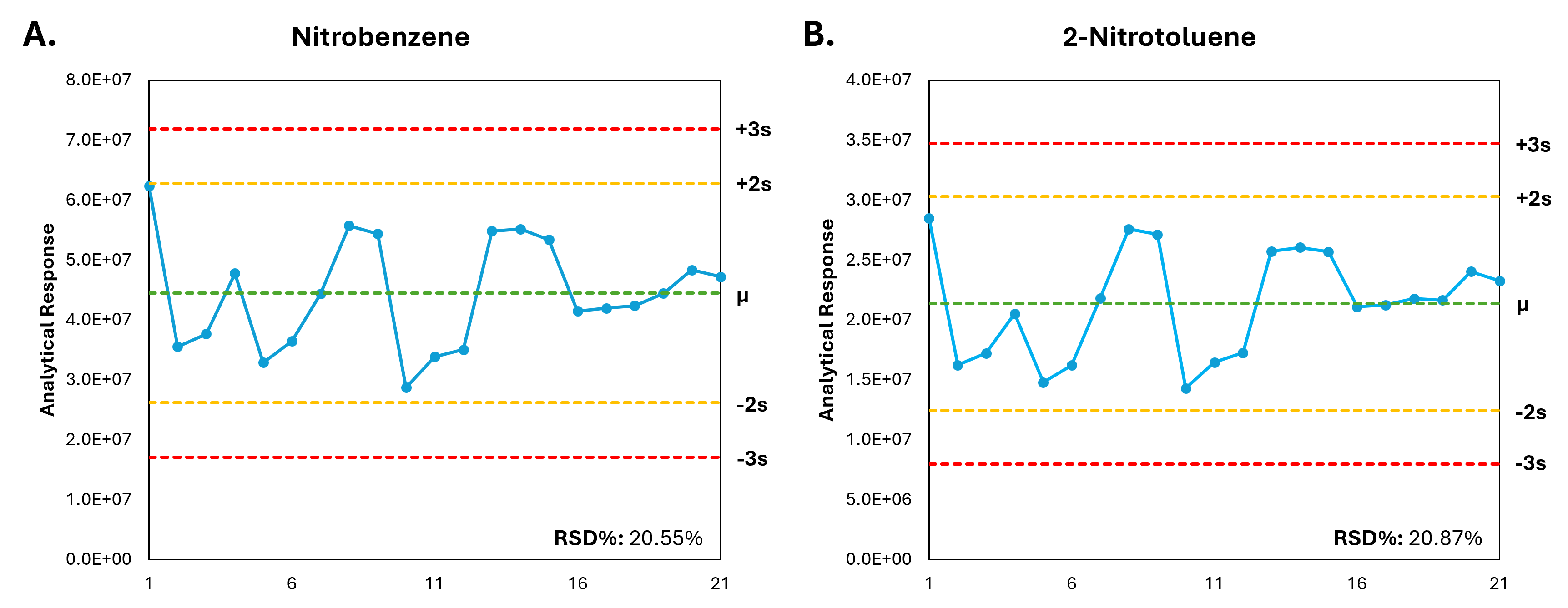

Supplement: Supplementary file 2 — Supporting File 2: jssc70442‐sup‐0002‐FigureS1.tif. [file JSSC-49-e70442-s002.tif]

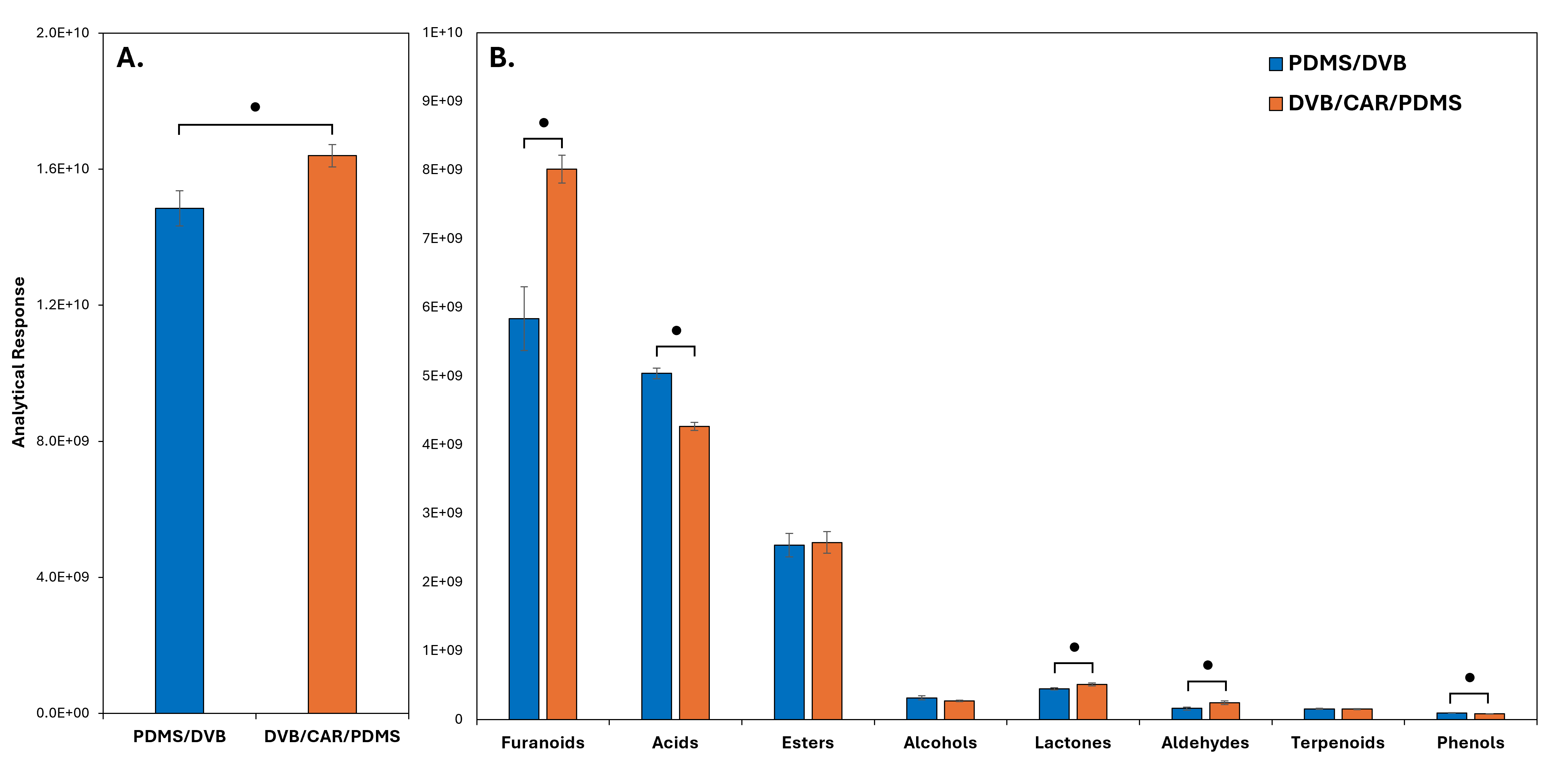

Supplement: Supplementary file 3 — Supporting File 3: jssc70442‐sup‐0003‐FigureS2.tif. [file JSSC-49-e70442-s003.tif]

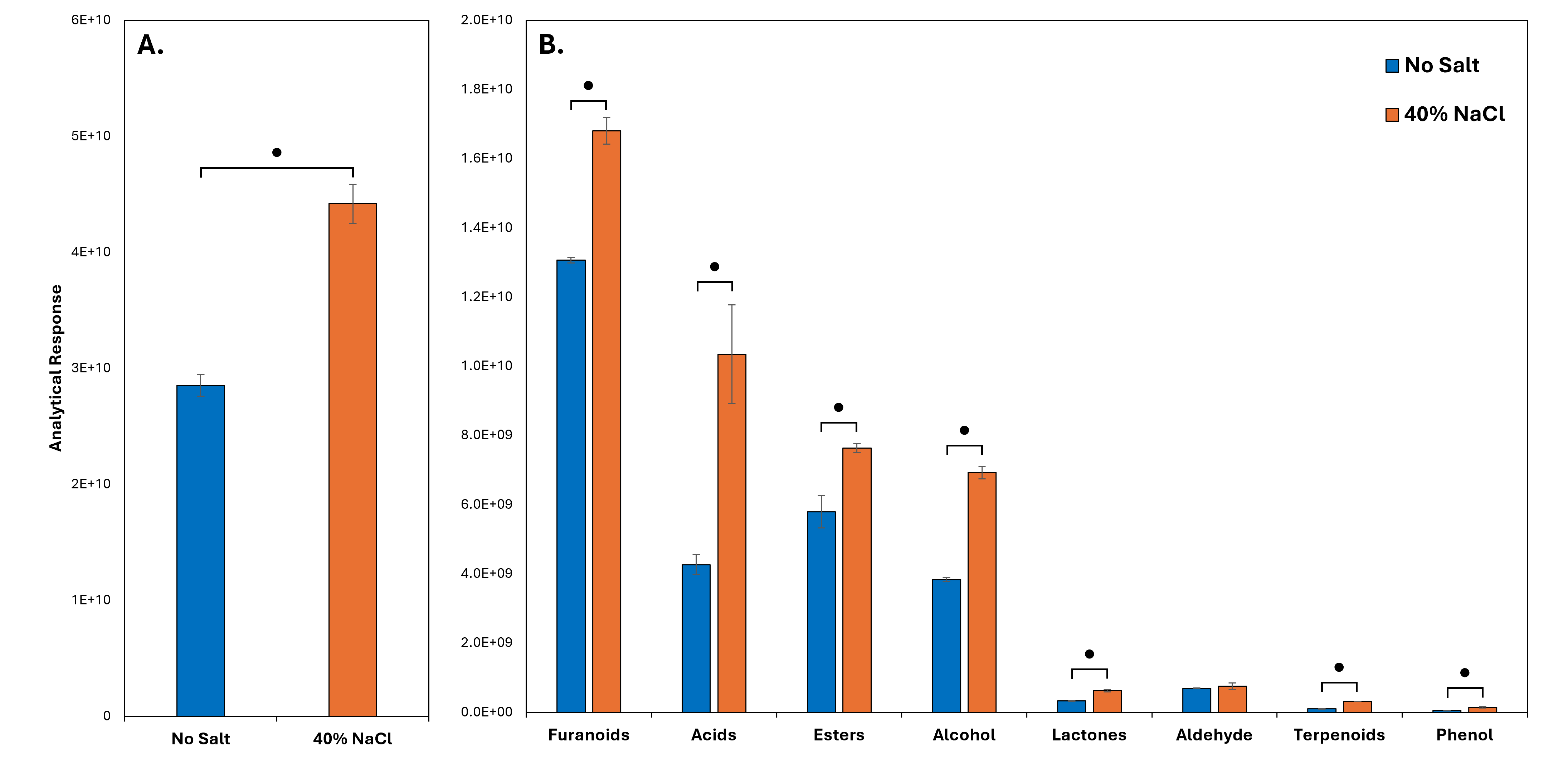

Supplement: Supplementary file 4 — Supporting File 4: jssc70442‐sup‐0004‐FigureS3.tif. [file JSSC-49-e70442-s001.tif]
